# Supplementary figures and images for: A voice-based biomarker for monitoring symptom resolution in adults with COVID-19: Findings from the prospective Predi-COVID cohort study
Source: PLOS Digit Health. 2022 Oct 20;1(10):e0000112. doi: 10.1371/journal.pdig.0000112 (PMC9931359; doi:10.1371/journal.pdig.0000112)

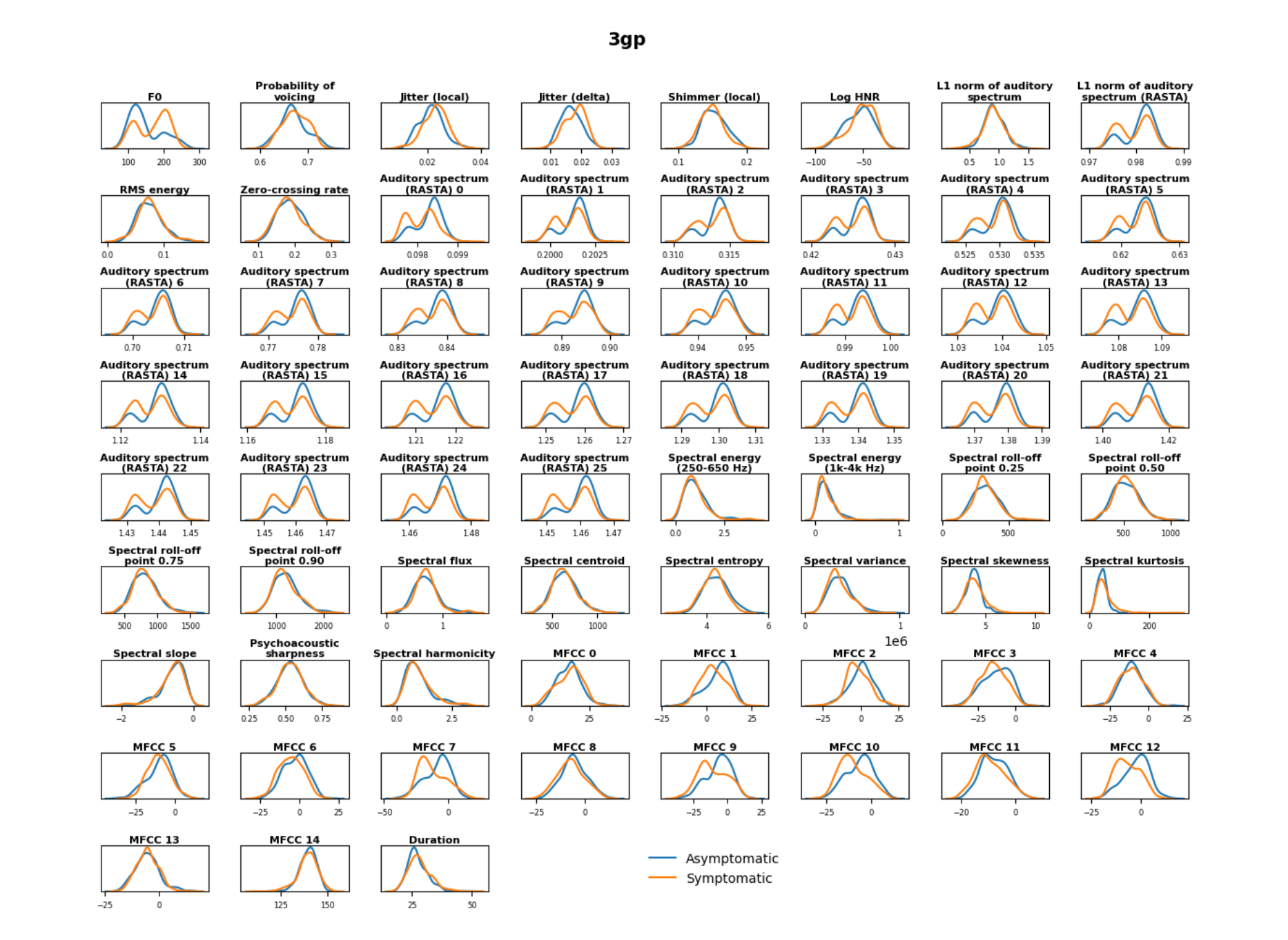

Supplement: S3 File — (TIFF) [file pdig.0000112.s003.tiff]

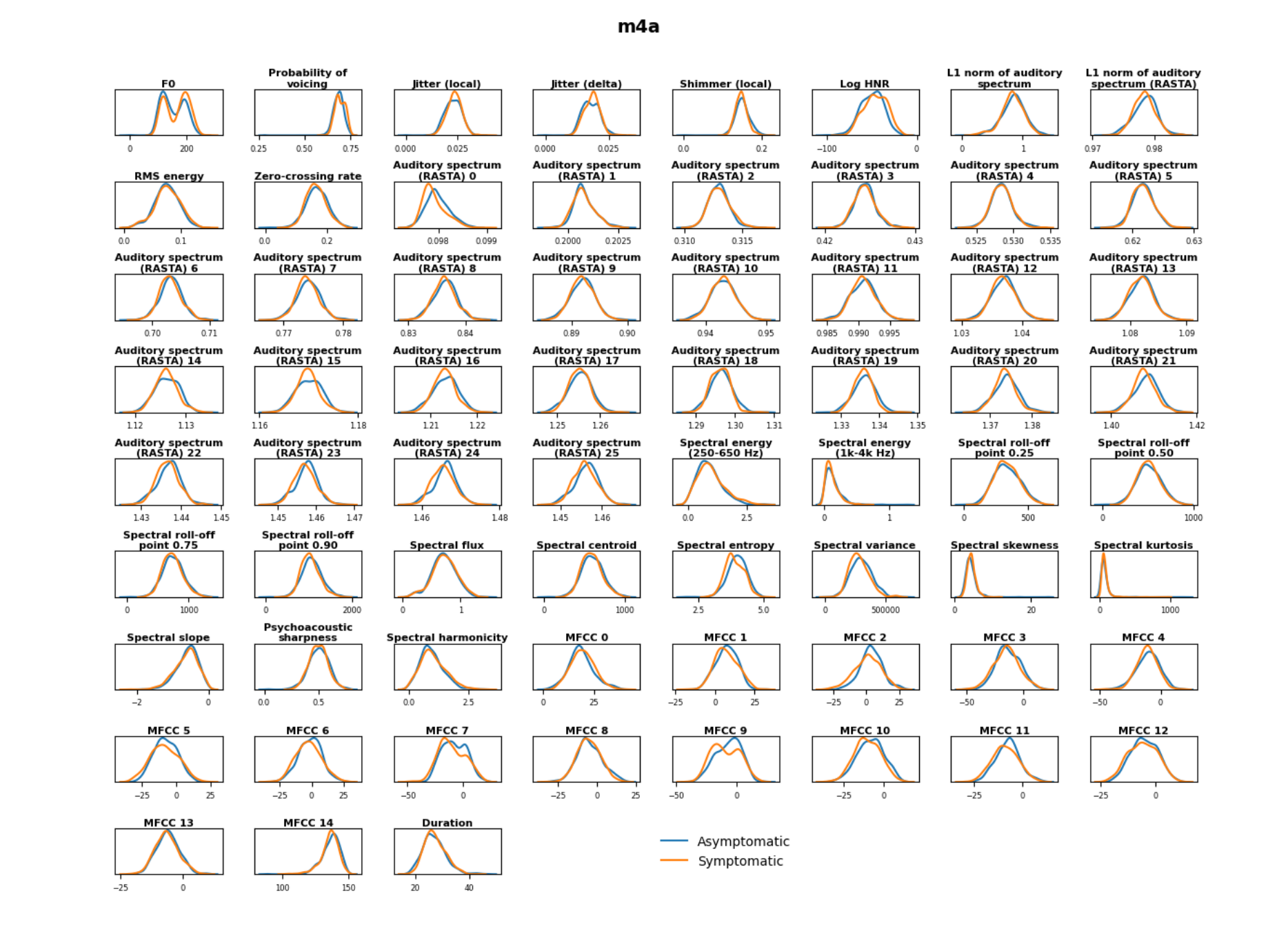

Supplement: S4 File — (TIFF) [file pdig.0000112.s004.tiff]
