# Supplementary material for: A voice-based biomarker for monitoring symptom resolution in adults with COVID-19: Findings from the prospective Predi-COVID cohort study
Source: PLOS Digit Health. 2022 Oct 20;1(10):e0000112. doi: 10.1371/journal.pdig.0000112 (PMC9931359; doi:10.1371/journal.pdig.0000112)
Supplement: Attachment — Submitted filename: Rebuttal Letter - PLOS DIGITAL HEALTH.docx [file pdig.0000112.s006.docx]

**Journal Requirements**:

1. Please amend your detailed Financial Disclosure statement. This is published with the article, therefore should be completed in full sentences and contain the exact wording you wish to be published.

**Response: The Financial Disclosure statement has been amended accordingly.**

State the initials, alongside each funding source, of each author to receive each grant.

**Response: Done.**

2. Please update your Competing Interests statement. If you have no competing interests to declare, please state: “The authors have declared that no competing interests exist.”

**Response: Done.**

3. We ask that a manuscript source file is provided at Revision. Please upload your manuscript file as a .doc, .docx, .rtf or .tex. If you are providing a .tex file, please upload it under the item type ‘LaTeX Source File’ and leave your .pdf version as the item type ‘Manuscript’.

**Response: Done.**

4. Please provide separate figure files in .tif or .eps format only and ensure that all files are under our size limit of 20MB. Please also ensure that all files are under our size limit of 20MB.

**Response: Done.**

**Reviewers' comments:**

Reviewer's Responses to Questions

1. Does this manuscript meet PLOS Digital Health’s

Is the manuscript technically sound, and does the data support the conclusions? The manuscript must describe methodologically and ethically rigorous research with conclusions that are appropriately drawn based on the data presented.

Reviewer #1: Yes

Reviewer #2: Yes

--------------------

2. Has the statistical analysis been performed appropriately and rigorously?</font>

Reviewer #1: Yes

Reviewer #2: Yes

Reviewer #3: Yes

--------------------

3. Have the authors made all data underlying the findings in their manuscript fully available (please refer to the Data Availability Statement at the start of the manuscript PDF file)?

PLOS Data policy requires authors to make all data underlying the findings described in their manuscript fully available without restriction, with rare exception. The data should be provided as part of the manuscript or its supporting information, or deposited to a public repository. For example, in addition to summary statistics, the data points behind means, medians and variance measures should be available. If there are restrictions on publicly sharing data—e.g. participant privacy or use of data from a third party—those must be specified.

Reviewer #1: Yes

Reviewer #2: Yes

Reviewer #3: Yes

--------------------

4. Is the manuscript presented in an intelligible fashion and written in standard English? PLOS Digital Health does not copyedit accepted manuscripts, so the language in submitted articles must be clear, correct, and unambiguous. Any typographical or grammatical errors should be corrected at revision, so please note any specific errors here.

Reviewer #1: Yes

Reviewer #2: Yes

Reviewer #3: Yes

--------------------

5. Review Comments to the Author. Please use the space provided to explain your answers to the questions above. You may also include additional comments for the author, including concerns about dual publication, research ethics, or publication ethics. (Please upload your review as an attachment if it exceeds 20,000 characters)

**Reviewer #1**: The paper presents a machine-learning based approach for the automatic identification of the presence of COVID-19 symptoms from the voice signal, proposing a “biomarker” which ultimately is the calibrated probability of being symptomatic. A pool of COVID-positive patients were recruited (Predi-COVID cohort), and vocal recordings were collected along with self-reported symptomatology questionaries, throughout a year-long period. There is heterogeneity of language and demographics within the subjects. A pipeline was built, starting from audio pre-processing followed by a wrapper-based feature selection and multiple classifiers, the better of which was chosen as the final model, whose calibrated outputs bring the “biomarker”. The test is independently divided between Android and iOS-based recording devices.

The paper is well written and coherently organized, although there’s an abundance of sections. Moreover, the building and usage of a new dataset and the search for symptomatology are a strong contribution to the originality of the study.

**Response: We thank Reviewer 1 for this positive feedback.**

However, there is a number of weak points that affect the generality of the work.

Especially when building custom datasets, which are often small, a peculiar attention to the cleanliness and a rigorous methodology should be employed. The recording conditions are not explained with sufficient detail. The main point that should be addressed with regards to the recordings delas with the source audio. In the “Data Analysis” section, the format of the files is stated to be 3GP for Android and M4A for iOS. Both of these extensions employ an AAC coding for audio signals, which is inherently lossy. This often leads to unsatisfactory and/or biased performances as well as loss of information for the audio analysis. An app is said to be used: since it manages the audio recording, we argue that it should record uncompressed/lossless audio. Additionally, no information is given on the compression parameters, sampling frequency and bit-depth of the collected audio.

**Response: We thank Reviewer 1 for the suggestion. A smartphone app was indeed used to collect the audio recordings in the study. The main objective of our approach is to derive vocal biomarkers that can be used in a real-life setting, meaning relying on the variety of smartphones available in the market. As such, we have decided to train the algorithms based on AAC coding recordings. We fully agree with Reviewer 1 that these formats induce a loss of information, but this is a necessary trade-off in anticipation of the future implementation in real life of such a digital health solution in practice where multiple devices are used. We have added a sentence in the manuscript to address Reviewer 1’s point.**

**Regarding the second comment, we have followed your recommendations and added elements to the manuscript on bit rates, and one now can read the following sentence in the manuscript, material and methods section: “First, audio files have all been converted into .wav files, using the ffmpy.FFmpeg() function by keeping the original sampling rate, i.e. 8kHz and 44.1kHz for 3gp and m4a respectively, and with 16-bit bit-depth. The compression ratio is around 10:1 for 3gp files and between 1.25:1 and 10:1 for m4a files.”**

The automatic selection of the recordings is interesting, although very briefly explained in the “Pre-processing” section. Some points could be stated for the whole pre-processing and feature extraction part:

1)	Some references or a concise discussion on the validity of the proposed selection methods could be beneficial, as it could be argued that “bad” recordings might have been left in if the selection methods weren’t sufficiently accurate.

**Response: We agree, we have now added a sentence in the discussion section to mention this possibility. Now we can read in the discussion: “Because the recordings were performed in real life, we have first cleaned and pre-processed the audio recordings and developed a pipeline to ensure that the vocal biomarkers training is as clean as possible, but we cannot completely rule out the possibility of having a few recordings of low quality.”**

2)	The pydub.effects.normalize function is stated to bring “a volume boost on the quiet parts”. Although the manual for the function states so, it is inherently wrong. This kind of normalization simply brings the peak of the signal to 0dbFS. The relative volume throughout different sections of the signal stays the same. Quiet parts are selectively raised in volume if compression is employed, or if normalization happens on smaller sections of the main signal, which appears not to be the case

**Response: We thank Reviewer 1 for raising this point. We took your comment into consideration and have now rephrased the initial sentence that could be confusing as such: “The pydub.effects finds the maximum volume of an audio segment, then adjusts the rest of the audio in proportion.”**

3)	Noise reduction is applied, using the logMMSE method. Using a noise reduction algorithm is a very delicate matter in audio analysis, as it inevitably changes and denatures the original data, possibly bringing artifacts not found in common speech signals, even when perceptual characteristics seem to improve. Therefore, a more thorough explanation and some proper referencing are needed in order to disclose the very algorithm used, the framework/library it has been employed with, and the reason why it should improve performance.

**Response: Thank you for bringing this up. Noise reduction is performed using the short-time spectral amplitude estimator that minimizes the mean-square error of the log spectrum (logMMSE), which is shown to result in a substantially lower residual noise level without affecting the voice signal substantially [see Y. Ephraim, D. Malah, Speech enhancement using a minimum mean-square error log-spectral amplitude estimator, IEEE Trans. Acoust. Speech Signal Process. Vol. 33, 443-445, 1985]. Further details and an appropriate reference are provided in the revised article. Although we were aware of the risk of distorting the signal and introducing artifacts in certain cases, this was a necessary trade-off due to the presence of noise in some recordings (either environmental noise or caused by the recording equipment), mainly caused by data collection in an uncontrolled environment and using devices of different quality.**

4)	It is not totally clear if there is a one-to-one correspondence between an audio recording of a patient and his questionaries. Does a patient record arbitrarily, whereas questionaries are (mostly) compulsory?

**Response: Thank you for the opportunity to clarify the study design. During the follow-up, the Predi-COVID participants were invited to report their symptoms and health every day for the first 14 days. In parallel, they received daily notifications to perform the standardized audio recordings. Both tasks were optional for the participants. For the analysis, we have simply analyzed the data when both an audio recording and a questionnaire were available for a given day to ensure that the audio features in the voice were associated with the reported symptoms. This information is mentioned in Figure 1 and mentioned in the text as follows (Methods section -> Study Design and Population paragraph): “Participants were asked to answer these questionnaires as often as possible but were free to skip them if they felt too ill or if symptoms did not materially change from one day to the other.” And “...with available audio recordings at any time point in the first two weeks of the follow-up and who had filled in the daily questionnaire on the same day as the audio recordings”.**

5)	A better explanation behind the choice of adding the MFCC0 features, and on their nature as well, could be beneficial.

**Response: Thank you for this comment. We have decided to add the zero-order MFCC as it represents the average of log-energy, which is commonly used in speech recognition. Applying the logarithm to the computation of energy mimics the dynamic variation in the human auditory system, making the energy less sensitive to input variations that might be caused by the speaker moving closer or further from the microphone. This has now been clarified in the manuscript in the Material and Methods section.**

The machine-learning part has some strong points, such as the use of many classifiers and steps, but needs some clarification and a more coherent explanation of some of the choices.

**Response: Thank you for this comment. We have chosen 4 commonly used methods in the field of machine learning, which have previously been used in the field of audio analysis. First, Support Vector Machine (SVM) is a widely used machine learning method for audio classification (see for instance doi: 10.1186/1687-4722-2012-16). SVM is based on the identification of a hyperplane that distinctly classifies the data points. Then we added the bagging approach, an ensemble method, to reduce the variance of a single model. (doi.org/10.1155/2015/209814,doi.org/10.1007/BF00058655). Random forest (RF) is another ensemble method that fits many decision trees on various sub-samples of the dataset and uses averaging to improve the predictive accuracy and control over-fitting (doi.org/10.1023/A:1010933404324, DOI: 10.1007/978-3-642-31537-4_13). We compared RF with the classical decision trees with bagging which is better to use when the classifier is not stable (**[**doi.org/10.4028/www.scientific.net/AMM.462-463.225**](http://doi.org/10.4028/www.scientific.net/AMM.462-463.225)**). These elements have now been integrated into the main manuscript.**

In the Feature Selection section, the theory behind the RFE is not clearly explained, no references are present and the first sentence should be re-written. Additionally, it is not specified that it is a wrapper-based method and there is no clear explanation for the way the number of features is reached using Random Forest classifiers.

**Response: Thank you for the comment. A more in-depth explanation of RFE is now provided in the revised manuscript, as given below:**

**“Recursive Feature Elimination (RFE) is a wrapper-based feature selection method, meaning that the model parameters of another algorithm (e.g. SVM or Random Forest) are used as criteria to rank the features, and the ones with the smallest rank are iteratively eliminated. In this way, the optimal subset of features is extracted at each iteration, showing that the features with the highest rank (eliminated last) are not necessarily the most relevant individually [Guyon, I., Weston, J., Barnhill, S. et al. Gene Selection for Cancer Classification using Support Vector Machines. Machine Learning 46, 389–422 (2002)]. We used Random Forest as a core estimator and the optimal number of selected features was determined using a grid search in the range [100, 150, 200, 250, 300]. The optimal number of features is provided in the subsection “Best predictive model” of the “Results” section.”**

However, the most crucial point in the machine-learning section is the training-test split and the cross-validation throughout all the steps. What appears from the manuscript is that a 5-fold cross-validation was independently used within every different step, starting from the feature selection. However, since the 5 folds of in Classification step will be different, the features selected beforehand will be based on samples that may be the same that are now in the testing fold. This generates an inherent bias. Technically, the test data should never endure a feature selection, not even partly. Nevertheless, in section “Derivation of the digital biomarker” a “distribution in the test dataset” is mentioned. Which test dataset is it referring to? Perhaps the cross-validation happened only once, before the Feature Selection, as Figure 1 may suggest; this is not clear.

**Response: Thank you very much for giving us the opportunity to clarify this important aspect. We have added a complementary analysis strategy to compare with the one previously provided. Two strategies were now tested to assess the model performances. The first strategy was to perform a 5-fold cross-validation, in order to have more information, but potentially based on partially already seen data. The second strategy was the assessment of the model performances only on the test dataset corresponding to the training dataset where the highest performances were obtained to avoid the use of partially seen data in the test dataset. We have modified Table 3 accordingly. The numbers outside the bracket now indicate the mean values from the 5-fold cross-validation across the whole dataset. In bracket is the performance on the test dataset where the model was trained on a separate training dataset. The results did not materially change between the two strategies so we have continued to use the performances from the best model identified with the 5-fold cross-validation to derive the corresponding vocal biomarkers. The corresponding sentences in the section “Derivation of the digital vocal biomarker”**

Several other points should be clarified, justified or corrected:

1)	In the “Classification model selection and evaluation” it is said that “We evaluated the significance of the cross-validated scores of the final model with 1000 permutations.”. The sentence is unclear and the methodology should be explained.

**Response: Thank you for your comment. We evaluated the significance of the cross-validated score of the final model with 1000 permutations (Ojala and Garriga. Permutation Tests for Studying Classifier Performance. DOI: 10.1109/ICDM.2009.108). Briefly, we permuted targets 1000 times to generate randomized datasets and calculated the cross-validated scores on each randomized dataset. The p-value represented the fraction of randomized datasets where the SVM model performed as well or better than the original data. The p values were < 0.001 for all the measures of the SVM model. It has now been further explained in the manuscript.**

2)	Since it is (understandably) stated as the most reliable indicator, a more thorough explanation would be needed for the MCC, as well as referencing. It would also be beneficial to present its formula, possibly in terms of false positives (FP), false negatives (FN), etc. Moreover, there is a “T” missing in Matthews’ name.

**Response: Thank you. The typo was corrected for “Matthews” and we have added a more detailed explanation of the MCC in the Methods section -> Classification model selection and evaluation. However, we have preferred not to mention its formula, but rather reference a methodological paper for those willing to know more about it and why this index might be more reliable than the others (*Chicco, D., Tötsch, N. & Jurman, G. The Matthews correlation coefficient (MCC) is more reliable than balanced accuracy, bookmaker informedness, and markedness in two-class confusion matrix evaluation. BioData Mining 14, 13 (2021). https://doi.org/10.1186/s13040-021-00244-z*).**

3)	The MCC is also the only indicator left out in the “Best predictive model” section.

**Response: Indeed, it has now been corrected. We can now read: “The MCC was 0.70, F1-score was 0.85, precision 0.85, and recall 0.85. For iOS devices, the SVM model had an AUC of 0.85, and balanced accuracy of 0.77. The MCC was 0.54, F1-score was 0.77, precision 0.77, and recall 0.77.”**

4)	At the end of the same section, the calibrations are deemed as “good” because of their Brier scores. However, such scores are not explained or referenced. A Brier score of 0.09 means a correct prediction with a 70% certainty, so what does “good” exactly mean in this scenario?

**Response: We thank Reviewer 1 for the opportunity to clarify this sentence. We have now added the original reference for the Brier Score in the Methods section -> Classification model selection and evaluation paragraph. We agree with Reviewer 1 that the phrasing of the Brier Score interpretation can be misleading. Our aim was to state that the Brier scores were low, so we have decided to remove any subjective interpretation such as “good calibration” in the text.**

The meta-analysis is also very hasty and lackluster. There is a brief digression on cough-based studies on COVID, and then it’s just stated that “no similar work to ours, i.e. focusing on individuals with COVID-19, has been previously reported. No comparison was possible with potential other models based on voice recordings rather than cough …”. However, this is definitely not true, as there is a handful of studies dealing with speech (sometimes associated with cough too) for COVID-19. The main ones that should be assessed are:

1)	Robotti, C, Costantini, G et al. : Machine Learning-based Voice Assessment for the Detection of Positive and Recovered COVID-19 Patients (Journal of Voice, DOI:<https://doi.org/10.1016/j.jvoice.2021.11.004>)

2)	Pinkas, G, Karny, Y et al. : SARS-CoV-2 detection from voice. (IEEE Open J Eng Med. 2020)

3)	Shimon, C, Shafat, G et al.: Artificial intelligence enabled preliminary diagnosis for COVID-19 from voice cues and questionnaires. (J Acoust Soc Am. 2021)

In the end, despite the novelty of the work and the undying necessity for quality research on COVID-19, we argue that this paper needs major revisions. A more thorough analysis and justification for the quality of the audio should be presented, as well as some clearance in the machine-learning section, and definitely a more extensive literature analysis.

**Response: We are thankful for the comment from Reviewer 1 on the discussion section of our work, as well as for the suggestions on additional references to consider. We would like to mention that reference n°1, by Robotti et al. was not published yet at the time of submission of the present work. We were not aware of this initiative, but we have now expanded the discussion, and used this opportunity to include this work in our references and discuss it. References 2) and 3) aimed at detecting (or diagnosing) COVID-19 based on voice or voice coupled with questionnaires. We have followed Reviewer 1’s suggestions and integrated them into the present paper, even though they do not allow us to compare our results directly against theirs, as our objective is to classify, exclusively in people with COVID-19, the symptomatic status based on voice.**

**Reviewer #2**: Review of “a Voice-based Biomarker for Monitoring Symptom Resolution in Adults with Covid-19: Findings from the Prospective Predi-Covid Cohort Study” for PLOS

This paper sets out to derive a digital vocal biomarker to monitor COVID-19 symptom resolution. The authors used data from a prospective COVID study which included voice recordings from both symptomatic and asymptomatic participants who tested positive for COVID. Artificial intelligence models were trained to detect the resolution of symptoms.

The paper would be improved by including additional narrative clarifying the methodology. PLOS Digital Health includes studies from a variety of disciplines, and not everyone will have an extensive background in artificial intelligence modeling or handling digital voice recordings. Please include a list of the acronyms and their meanings.

**Response: Thank you for this comment, which was in line with a similar suggestion from another Reviewer. We have followed the suggestion and have now expanded the material and methods section to facilitate the understanding of the methodology and pipeline by non experts.**

Please include the total number of audio recordings and the number of symptomatic cases and asymptomatic cases in the text in the final paragraph under “methods.”

**Response: Thank you for this suggestion, but as these numbers are derived from our methodological pipeline, and based on our inclusion and exclusion criteria, we have preferred to keep them in the Results section. In the first paragraph, “Study participants' characteristics”, we can already read that 272 participants were included and that the total number of audio recordings analyzed was 1775, distributed as 1049 from symptomatic individuals at the time of the recording, and 726 asymptomatic individuals at the time of recording.**

In developing the list of COVID-19 related symptoms that were included in self-report questionnaires, did the authors consider “Flu-pro plus”? This is widely used in the United States.

**Response: Thank you for your suggestion and for mentioning the “Flu-pro plus” tool. For the present work, we used items adapted from the International Severe Acute Respiratory and Emerging Infection Consortium (ISARIC) eCRF to identify the most frequent Covid-19 related symptoms. The detailed Predi-COVID study protocol can be found here: Fagherazzi G, Fischer A, Betsou F, et alProtocol for a prospective, longitudinal cohort of people with COVID-19 and their household members to study factors associated with disease severity: the Predi-COVID study. BMJ Open 2020;10:e041834. doi: 10.1136/bmjopen-2020-041834.**

On page 4 under the section “pre-processing, the sentence beginning “First, audio files…” appears to be missing something in parenthesis. The sentence on noise reduction appears to be incomplete.

**Response; Thank you for your comment. We have checked the corresponding paragraph. The parentheses are part of the functions we used. The functions are named ffmpy.FFmpeg() and pydub.effects.normalize(). We have removed the parentheses to avoid confusion. The sentence on noise reduction has been corrected as well.**

On page 5, “Feature extraction,” please explain more about why you did this. This should be clear to those unfamiliar with sound processing and artificial intelligence modeling.

**Response: Thank you for this comment. Feature extraction is performed to reduce the dimensionality and extract meaningful information from raw audio signals in a compact and expressive form that could be further processed by a machine learning algorithm. The comment on this is added in the revised article in the Methods Section -> Feature selection paragraph.**

The sentence starting “We compared the distribution…” talks about the arithmetic mean of symptomatic versus asymptomatic samples but also discusses separating android versus iOS audio in one confusing sentence. It would be clearer if these two concepts were described separately.

**Response: Done, thank you.**

On page 5, “classification model selection and evaluation,” please explain the different models and why you would choose to test all four.

**Response: Thank you for your comment. We decided to integrate these state-of-the-art machine learning techniques to identify the best possible pipeline to identify the vocal biomarkers. Following a recommendation from another Reviewer, we have also now tested a deep learning model, named feedforward neural network, that has been added to the manuscript.**

On page 7, in the last sentence of the section entitled “comparison with the literature,” do you mean “other” instead of the word “rather”?

**Response: Indeed, the typo is now corrected. Thank you.**

On Table 1, does the table include only symptomatic participants? It’s confusing, because you mentioned you started out with 272 study participants, but I could not find the number of symptomatic versus asymptomatic participants.

**Response: Thank you for this opportunity to clarify the content of Table 1. We agree with Reviewer #2, this may be confusing. Table 1 is presenting data only for participants who had reported at least one symptom during the 14 days of follow-up. The legend of Table 1 has been modified accordingly to avoid confusion.**

On table 3, please include a key with a brief description of the machine learning algorithm, and the acronyms. On this table, it appears that “cases” means the number of voice recordings, not the number of actual participants. It would be good to clarify this.

**Response: Thank you for this suggestion. We have now added a brief description in the main manuscript for each model presented in Table 3. We have also reworded the sentences with “cases”. Now, we can read for instance "Android devices / 3gp: 449 voice recordings from symptomatic individuals / 244 voice recordings from asymptomatic individuals”.**

In your discussion section, you might consider including possible future directions. You did not have a COVID free control group. I wondered whether the voices of people who had not tested positive for COVID would be different from people who tested positive but were asymptomatic. It appears that you compared voice recordings of participants indicating symptoms with voice recordings of participants who did not indicate any symptoms. So, over the course of time, would a person have been classified as “asymptomatic” following the resolution of symptoms that were reported earlier? If that is correct, it might be also interesting to look at the same participant’s voice while he/she indicates symptoms with that same participant’s voice after the symptoms have resolved.

**Reviewer #3**: This is a clearly written, timeliness paper. To my point of view, its major strengths rely on the good data availability and reproducibility, and the large specific covid-19 symptom database.

However, the paper have some major drawbacks, outlined next:

1. The database is large enough to perform state-of-the-art deep learning-based classification algorithms. However, only some traditional algorithms were tested. Although DL algorithms shouldn't necessary perform better, they should be included in the study.

**Response: Thank you, we have followed your recommendation and included a deep learning model in our analysis strategy. The final results did not change, as they did not outperform the SVM models.**

2. The authors extract a huge number of features from OpenSmile (6472), and then they perform a feature selection using a Recursive Feature Elimination method. However, there is not detailed description of which voice features were extracted and selected, nor an analysis of the relevance of the extracted features for the classification. Are the best features more related to prosodic, only acoustic, dynamic features? Such analysis would be crucial for a better understanding of a speech-based biomarker model.

**Response: Thank you for this comment. We have followed the Reviewer’s suggestion and have now added a column in the Supplementary Online Material 4 with the type of features selected for each audio format. For 3gp, we have observed that the selected features were mainly coming from the spectral (53%) domain, followed by cepstral (37%), prosodic (8%), and voice quality features (2%). We observed a similar trend for m4a, as we have observed that the selected features were mainly coming from the spectral (59%) domain, followed by cepstral (29%), prosodic (9%), and voice quality features (3%). This is now mentioned in the Results section.**

3. Apart from the large database collection with its specificities, the current study does not represent a novelty with respect to the start of the art. The techniques used are not novel, and the discussion of what features are relevant as biomarker, which would be a good contribution to the paper, are not presented. The authors mention that "The most promising approach so far has been proposed by the MIT, as they achieved an elevated sensitivity (98.5% in symptomatic and 100% in asymptomatic individuals) and specificity (94.2% in symptomatic and 83.2% in asymptomatic individuals).". Which is the study they refer to? This approach is not properly referenced. Also, what are the main differences with respect to MIT's study that could make this contribution novel?

**Response: Thank you for raising this point. We would like to clarify that our aim was not to develop new methodological approaches but rather to apply existing models to a clinically relevant use case. Consequently, we used state-of-the-art approaches to train our vocal biomarkers. We have added the reference related to the MIT study. However, as already mentioned in the article, their aim was different. Based on cough, they want to detect the presence of COVID-19. They did not aim to train a vocal biomarker for monitoring the symptom status in people with confirmed COVID-19, as we did.**

4. One of the limitations mentioned by the authors is that "Our vocal biomarker is mostly based on French and German voice recordings and as audio features may vary across languages or accents, our work will have to be replicated in other settings and populations." The overcome this, the experiments could have been performed separately by languages, so that it would be possible to analyse the language dependency to the biomarker.

**Response: Thank you very much for this suggestion. As shown in Table 2, there is an over-representation of French language in the dataset. Due to the limited number of audio recordings available in the other languages (German, English or Portuguese), it is not possible to train separate models with reasonable power. This important question might be addressed in subsequent work.**

Minor remark:

- "350ms" --> "350 ms"

**Response: Done, thank you**

--------------------

6. PLOS authors have the option to publish the peer review history of their article

If published, this will include your full peer review and any attached files.<br><br>

Do you want your identity to be public for this peer review? If you choose “no”, your identity will remain anonymous but your review may still be made public.

For information about this choice, including consent withdrawal, please see our Privacy Policy.

Reviewer #1: No

Reviewer #2: No

Reviewer #3: No

--------------------

Comments to the Author

1. Does this manuscript meet PLOS Digital Health’s publication criteria? Is the manuscript technically sound, and do the data support the conclusions? The manuscript must describe methodologically and ethically rigorous research with conclusions that are appropriately drawn based on the data presented.</font>

Reviewer #3: Yes

--------------------

[NOTE: If reviewer comments were submitted as an attachment file, they will be attached to this email and accessible via the submission site. Please log into your account, locate the manuscript record, and check for the action link "View Attachments". If this link does not appear, there are no attachment files.]

While revising your submission, please upload your figure files to the Preflight Analysis and Conversion Engine (PACE) digital diagnostic tool, PACE helps ensure that figures meet PLOS requirements. To use PACE, you must first register as a user. Registration is free. Then, login and navigate to the UPLOAD tab, where you will find detailed instructions on how to use the tool. If you encounter any issues or have any questions when using PACE, please email PLOS at figures@plos.org. Please note that Supporting Information files do not need this step.
