# Supplementary material for: A voice-based biomarker for monitoring symptom resolution in adults with COVID-19: Findings from the prospective Predi-COVID cohort study
Source: PLOS Digit Health. 2022 Oct 20;1(10):e0000112. doi: 10.1371/journal.pdig.0000112 (PMC9931359; doi:10.1371/journal.pdig.0000112)
Supplement: Attachment — Submitted filename: Rebuttal Letter - PDH.docx [file pdig.0000112.s007.docx]

**Reviewers' comments**

**Reviewer's Responses to Questions**

**Comments to the Author**

1. If the authors have adequately addressed your comments raised in a previous round of review and you feel that this manuscript is now acceptable for publication, you may indicate that here to bypass the “Comments to the Author” section, enter your conflict of interest statement in the “Confidential to Editor” section, and submit your "Accept" recommendation.

Reviewer #1: All comments have been addressed

Reviewer #2: All comments have been addressed

--------------------

2. Does this manuscript meet PLOS Digital Health’s publication criteria? Is the manuscript technically sound, and do the data support the conclusions? The manuscript must describe methodologically and ethically rigorous research with conclusions that are appropriately drawn based on the data presented.

Reviewer #1: Yes

Reviewer #2: Yes

--------------------

3. Has the statistical analysis been performed appropriately and rigorously?

Reviewer #1: Yes

Reviewer #2: Yes

--------------------

4. Have the authors made all data underlying the findings in their manuscript fully available (please refer to the Data Availability Statement at the start of the manuscript PDF file)? PLOS Data policy requires authors to make all data underlying the findings described in their manuscript fully available without restriction, with rare exception. The data should be provided as part of the manuscript or its supporting information, or deposited to a public repository. For example, in addition to summary statistics, the data points behind means, medians and variance measures should be available. If there are restrictions on publicly sharing data—e.g. participant privacy or use of data from a third party—those must be specified.

Reviewer #1: Yes

Reviewer #2: Yes

--------------------

5. Is the manuscript presented in an intelligible fashion and written in standard English? PLOS Digital Health does not copyedit accepted manuscripts, so the language in submitted articles must be clear, correct, and unambiguous. Any typographical or grammatical errors should be corrected at revision, so please note any specific errors here.

Reviewer #1: Yes

Reviewer #2: Yes

--------------------

6. Review Comments to the Author. Please use the space provided to explain your answers to the questions above. You may also include additional comments for the author, including concerns about dual publication, research ethics, or publication ethics. (Please upload your review as an attachment if it exceeds 20,000 characters)

Reviewer #1: MINOR REVISION

The authors addressed most of the concerns with reasonable answers.

Several limitations on the scientific validity of the proposed study still emerge, especially on the quality of the proposed dataset and on the reproducibility of the methodology. However, the proposal is coherent and interesting enough for publication, with a suggested minor revision addressing the following points:

**Response: Thank you very much for your constructive and respectful feedback.**

-	In the response, the authors state that lossy audio is “a necessary trade-off in anticipation of the future implementation in real life of such a digital health solution in practice where multiple devices are used”. We politely disagree, as losing on audio quality on sources as diverse and complex as self-recorded voice could really hinder the quality of the measurements; on the other hand, it is feasible to provide an App or API that allows different devices to record lossless audio.

**Response: We thank Reviewer #1. We agree with this comment. Since our recordings were acquired in the compressed format, we have extended the Limitation subsection with additional information about the quality of recording constraints (please see the related response below).**

-	For the abovementioned reasons, it would be beneficial to clearly state the main limitations of this study for the readers to realize: lossy audio, sub-optimal recording conditions and lack of control (already mentioned in the Limitations section), non-homogeneous processing due to the .m4a compression and possible artifacts induced with the usage of logMMSE noise reduction.

**Response: We fully agree and have extended the limitation subsection with additional information about the potential sources of low quality of recordings, as given below.**

**“This work also has some limitations. First, our analysis only covers the discovery and internal validation phases of the vocal biomarker. Because the recordings were performed in real life, we have first cleaned and pre-processed the audio recordings and developed a pipeline to ensure that the vocal biomarkers training is as clean as possible, but we cannot completely rule out the possibility of having a few recordings of low quality. Potential sources of low quality of recordings include sub-optimal recording conditions in an uncontrolled environment, the use of lossy audio formats for data compression and the artifacts potentially introduced by noise reduction.”**

-	The explanation of the 1000 permutations for training models is still unclear: which “targets” are permuted? Please re-write it

**Response: We agree with Reviewer #1 and have rephrased the sentence about permutations accordingly to improve readability. Now it reads: “We evaluated the significance of the cross-validated scores of the final model with 1000 permutations. Briefly, we generated randomized datasets by permuting only the binary outcome of the original dataset 1000 times and calculated the cross-validated scores on each randomized dataset. The p-value represented the fraction of randomized datasets where the model performed as well or better than the original data.” We have also added the following reference in the manuscript to support our approach. Ojala and Garriga. Permutation Tests for Studying Classifier Performance. DOI: 10.1109/ICDM.2009.108.**

-	It is unclear to me why the results of the proposed references detecting COVID-19 from the voice are not comparable to those in the present study. “Symptomatic” COVID is of course the main aim when trying to pre-diagnose it from the voice. However, this is just a minor consideration.

We thank the authors for following our suggestions.

**Response: We thank Reviewer #1 for this opportunity to clarify our point here. We agree that the suggested references are close to our own work, but we prefer not to directly compare them with our work as the categories to be predicted in the previous papers and ours are not the same and have different clinical purposes as well. The suggested references aimed to develop voice-based COVID-19 screening tools and predict the COVID-19 status based on voice. Their corresponding classifiers aimed to predict the classes “SARS-CoV-2 positive” versus “SARS-CoV-2 negative”, regardless of the symptomatic status of individuals in the “SARS-CoV-2 positive” group. Some of them even demonstrated that their classifiers worked to predict SARS-CoV 2 positive status in asymptomatic individuals, such as Laguarta J, Hueto F, Subirana B. COVID-19 Artificial Intelligence Diagnosis Using Only Cough Recordings. IEEE Open J Eng Med Biol. 2020;1: 275–281.**

**In our work, we have exclusively worked with “SARS-COV-2 positive” individuals, all validated with a PCR test and we have to try to classify their symptomatic status based on voice, and as such, the comparison group is different. This difference can be summarized in the figure below.**

Reviewer #2: Excellent contribution. Thank you for your response to reviewer comments.

**Response: Thank you very much for your review.**

--------------------

7. PLOS authors have the option to publish the peer review history of their article. If published, this will include your full peer review and any attached files. Do you want your identity to be public for this peer review? If you choose “no”, your identity will remain anonymous but your review may still be made public.

For information about this choice, including consent withdrawal, please see our Privacy Policy.

Reviewer #1: None

Reviewer #2: No

--------------------

While revising your submission, please upload your figure files to the Preflight Analysis and Conversion Engine (PACE) digital diagnostic tool, https://secure-web.cisco.com/1_1NH6gCjTL4I3SgpsGHoe8mUYHI8BHREo4AiMyWykiwJHGpKJWMAkG2rtg1m9hYaO9ECcQ3mLIEU6e5_KqbaySzO86b0l3NfFE9PXWg0aZfabrt9ECpbG2rWFK-SPBxYLEU2H_WB86OCA5_UranP-ejhjfkSA5kYI4vZdK8KQuyrXOVRVqyqD2pFjptNNt1CAnAnVWZnBPsaL678a0kRCHnQbWqyVAJY42KOUPK17nUjIAFS8rQVsKYIK06tNJF7O1n-9frTdjSUf1jBb2dVqopIaMtsgNh4P2dUTkqM0Ri-LPFvQwNuMUuhaqQMRuutySsbfRr9znZHVxUZomsvcQ/https%3A%2F%2Fpacev2.apexcovantage.com%2F PACE helps ensure that figures meet PLOS requirements. To use PACE, you must first register as a user. Registration is free. Then, login and navigate to the UPLOAD tab, where you will find detailed instructions on how to use the tool. If you encounter any issues or have any questions when using PACE, please email PLOS at figures@plos.org. Please note that Supporting Information files do not need this step.

**Response: Done**
